# Natural Products and Their Bioactive Compounds: Neuroprotective Potentials against Neurodegenerative Diseases

**DOI:** 10.1155/2020/6565396

**Published:** 2020-02-14

**Authors:** Nur Shafika Mohd Sairazi, K. N. S. Sirajudeen

**Affiliations:** ^1^Faculty of Medicine, Universiti Sultan Zainal Abidin (UniSZA), Medical Campus, Jalan Sultan Mahmud, 20400 Kuala Terengganu, Terengganu, Malaysia; ^2^Department of Chemical Pathology, School of Medical Sciences, Health Campus, Universiti Sains Malaysia, 16150 Kubang Kerian, Kelantan, Malaysia; ^3^Department of Basic Medical Sciences, Kulliyyah of Medicine, International Islamic University Malaysia, Bandar Indera Mahkota, 25200 Kuantan, Pahang, Malaysia

## Abstract

In recent years, natural products, which originate from plants, animals, and fungi, together with their bioactive compounds have been intensively explored and studied for their therapeutic potentials for various diseases such as cardiovascular, diabetes, hypertension, reproductive, cancer, and neurodegenerative diseases. Neurodegenerative diseases, including Alzheimer's disease, Huntington's disease, Parkinson's disease, and amyotrophic lateral sclerosis are characterized by the progressive dysfunction and loss of neuronal structure and function that resulted in the neuronal cell death. Since the multifactorial pathological mechanisms are associated with neurodegeneration, targeting multiple mechanisms of actions and neuroprotection approach, which involves preventing cell death and restoring the function to damaged neurons, could be promising strategies for the prevention and therapeutic of neurodegenerative diseases. Natural products have emerged as potential neuroprotective agents for the treatment of neurodegenerative diseases. This review focused on the therapeutic potential of natural products and their bioactive compounds to exert a neuroprotective effect on the pathologies of neurodegenerative diseases.

## 1. Introduction

Neurodegeneration is the progressive dysfunction and loss of neuronal structure and function that resulted in neuronal cell death [1, 2]. Neurodegeneration occurs in various diseases affecting the central nervous system (CNS). The loss of specific populations of neurons related to the functional neuronal networks determines the clinical presentation of acute and chronic neurodegenerative diseases. Neurodegenerative disease is a general term for a range of neurological disorder which primarily affects neurons in the CNS that are characterized by the gradual loss of neurons in the CNS, leading to deficits in specific brain functions (memory, movement, and cognition) [3].

Acute neurodegeneration is a condition in which neurons are rapidly damaged and usually die in response to a sudden insult or traumatic event such as head injury, strokes, traumatic brain injury, cerebral or subarachnoid hemorrhage, and ischemic brain damage [4]. Meanwhile, chronic neurodegeneration is a chronic state in which neurons in the nervous system undergo neurodegenerative process that usually starts slowly and worsen over time with multifactorial causes, resulting in the progressive and irreversible destruction of specific neuron populations [3, 5–7]. The chronic neurodegenerative diseases include Alzheimer's disease, Huntington's disease, Parkinson's disease, and amyotrophic lateral sclerosis.

Various types of biological mechanisms have been associated with neurodegeneration including oxidative stress, neuroinflammation, excitotoxicity, mitochondrial dysfunction, abnormal protein misfolding and aggregation, and apoptosis [8–16]. These biological processes have been implicated in the progression and pathogenesis of neurodegenerative diseases. To date, extensive studies have attempted to elucidate the mechanism and potential therapeutic targets to combat neurodegenerative diseases. Therefore, neuroprotection strategies and relative mechanisms work best to prevent or delay the process of neurodegeneration through the interaction with the pathophysiological change process.

Natural products are known and employed since ancient times for their therapeutic properties. In recent years, biological activities, nutritional values, and potential health and therapeutic benefits of natural products and their bioactive compounds have been intensively explored and investigated. Within the past decades, many studies have reported the protective effect of natural products and its bioactive compounds against various diseases such as cardiovascular, diabetes, reproductive, cancer, and neurodegenerative diseases. Natural products have emerged as potential neuroprotective agents for the treatment of neurodegenerative diseases. This review focused on the therapeutic potential of natural products and their bioactive compounds to exert neuroprotective effects on the pathologies of neurodegenerative diseases.

## 2. Neurodegeneration and Neurodegenerative Diseases: Mechanisms and Potential Therapeutic Targets

Neurodegenerative diseases, such as Alzheimer's disease, Huntington's disease, Parkinson's disease, and amyotrophic lateral sclerosis, are a group of disorders that are characterized by progressive and specific loss of cell in specific vulnerable neuronal populations of the CNS [6, 17]. Alzheimer's disease is an age-related and chronic, progressive neurodegenerative disease, which is associated with memory and cognitive impairments and behavioral changes. It is characterized by two major neuropathological hallmarks: (i) the formation and deposition of the extracellular amyloid-beta (A*β*) plaques and (ii) the protein accumulation of intracellular hyperphosphorylated tau proteins known as neurofibrillary tangles in the brain [18].

Parkinson's disease is a chronic and progressive neurodegenerative disease caused by a progressive loss of dopaminergic nigrostriatal neurons and diminishes the motor function, including resting tremor, postural imbalance, bradykinesia, and muscular rigidity [19]. The neuropathological hallmark of Parkinson's disease is the accumulation of intracellular protein aggregates, Lewy bodies, and Lewy neurites, which are predominantly formed of misfolded and aggregated forms of the presynaptic protein alpha (*α*)-synuclein and the progressive loss of dopaminergic nigrostriatal neurons [19].

Another progressive neurodegenerative disease, amyotrophic lateral sclerosis is characterized by progressive degeneration and death of upper and lower motor neurons, resulting in paralysis and death from respiratory failure. The mechanisms that underlie amyotrophic lateral sclerosis remain unknown, but several factors have been considered including genetic factors, excitotoxicity, oxidative stress, autoimmune response, impaired axonal transport, neurofilament aggregation, environmental factors, and mitochondrial dysfunction [20]. Amyotrophic lateral sclerosis is associated with mutation in the gene that produces the copper/zinc superoxide dismutase-1 (SOD1) enzyme.

Meanwhile, Huntington's disease is characterized pathologically by excessive dopaminergic activity and diminished gamma-aminobutyric acid (GABA) functions in the basal ganglia and clinically by abnormal movements, psychiatric disturbance, and cognitive deficits [21]. It is caused by a trinucleotide repeat expansion of the nucleotides cytosine, adenine, and guanine (a CAG expansion) in the Huntingtin (HTT) gene, located at the short arm of chromosome 4 [22].

**Figure 1:**
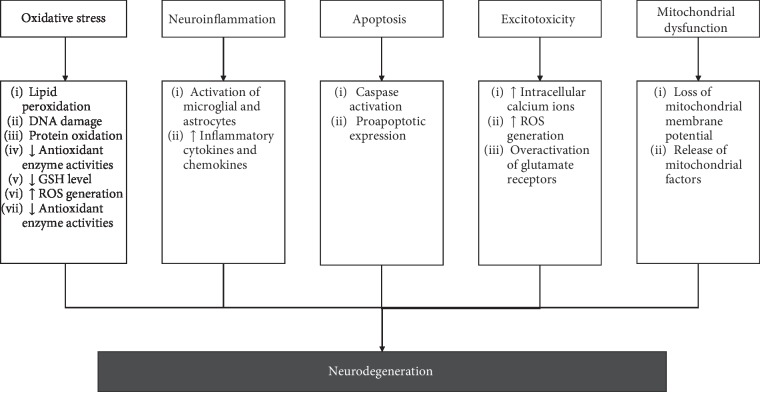
Various types of mechanisms have been associated with the neurodegeneration that implicated in the progression and pathogenesis of neurodegenerative diseases.

Various types of biological processes have been implicated in the progression and pathogenesis of neurodegenerative diseases, including oxidative stress, neuroinflammation, excitotoxicity, mitochondrial dysfunction, and apoptosis [8–16] (refer Figure 1).

Oxidative stress plays a contributory role in the pathophysiology of common neurodegenerative diseases [9–11]. The imbalance of the production of reactive oxygen species (ROS) and insufficient antioxidant defense capacity, causing oxidative stress to occur, results in cellular damage, DNA repair system impairment, and mitochondrial dysfunction. These will accelerate the neurodegenerative process and progression of neurodegenerative disease.

Moreover, it has been postulated that neuroinflammation may also play a role in the pathophysiology of neurodegenerative diseases [13, 23–25]. Neuroinflammation is an inflammatory process that involved in both innate and adaptive immune system in the CNS and has been associated with neurodegeneration. The mechanisms of neuroinflammation could contribute to the development of the normal brain and the neuropathological events. In the CNS, microglia are the major component of innate immune defense. Following the pathological changes within the nervous system, microglia rapidly acquired the morphology changes and activated microglia secrete several inflammatory mediators, such as cytokines, chemokines, and cytotoxic molecules (cyclooxygenase-2 (COX-2), ROS, glutamate, and prostaglandins). These inflammatory mediators will trigger astrocytes to induce secondary inflammatory or growth factor repair response as well as trigger neurons to respond for its survival.

Another biological process, excitotoxicity may also involve in the pathogenesis of neurodegenerative diseases [7]. It is defined as the pathological process of neuronal death caused by the over- or prolonged activation of glutamate receptors by excitatory amino acids or excitotoxins in the CNS [5]. Excitotoxins which bind to the glutamate receptors, as well as pathologically high levels of glutamate release, can cause excitotoxicity by allowing rapid entry of calcium ions (Ca^2+^) to enter the cell. Ca^2+^ influx into cells activates several Ca^2+^-dependent enzymes, including phospholipases, lipases, endonucleases, xanthine oxidase, protein phosphatases, proteases, protein kinase, and inducible nitric oxide synthase (iNOS). These enzymes go on to damage cell structures such as components of the cytoskeleton, membrane, and DNA [5, 26–30]. The excessive Ca^2+^ influx could also result in ROS production, mitochondrial dysfunction, oxidative stress, and inflammatory responses. These processes ultimately lead to neuronal cell death.

Neuronal apoptosis also plays a role in the neurodegenerative process. It is a highly regulated form of cell death and characterized by cell shrinkage, chromatin condensation, DNA fragmentation, and membrane cell death. This energy-dependent process requires ATP for protein synthesis and signal activation [31]. Apoptosis is a complex process which is triggered by intrinsic and extrinsic signal. The extrinsic pathway involves the activation of death receptors upon ligand binding and downstream signaling through cascade of protein-protein interactions. Meanwhile, the intrinsic pathway involves the release of proapoptotic factors located in the mitochondrial intermembrane space via mitochondrial permeability transition into cytosol and subsequently triggers caspase-dependent apoptosis or caspase-independent apoptosis (see [32]).

Mitochondria are the site of oxidative phosphorylation and cellular respiration and play a role in maintaining a low concentration of Ca^2+^ in the cytosol [28]. Excessive uptake of Ca^2+^ and generation of ROS cause the collapse of mitochondrial membrane potential and the opening of mitochondrial permeability transition pores [31]. The opening of mitochondrial permeability transition pores causes swelling of the mitochondrial matrix, which result in mitochondrial uncoupling, the rupture of the mitochondrial outer membrane, and the release of mitochondrial factors (cytochrome-c and apoptotic-inducing factor) located in the mitochondrial intermembrane space via mitochondrial permeability transition pores into cytosol [29, 32]. In caspase-dependent mechanism, cytochrome-c binds to apoptotic protease-activating factor-1 and procaspase-9 to form apoptosome complex and the activation of caspase-3 pathway. The activation of caspase is responsible for the activation of apoptotic neuronal death. This leads to the cleavage of essential cellular substrates such as poly (ADP-ribose) polymerase-1 (PARP-1). These alterations in the mitochondrial function could be an early event prior to neuronal cell death. In caspase-independent mechanism, apoptotic-inducing factor translocates to the nucleus and induces DNA fragmentation and chromatin condensation [32].

**Figure 2:**
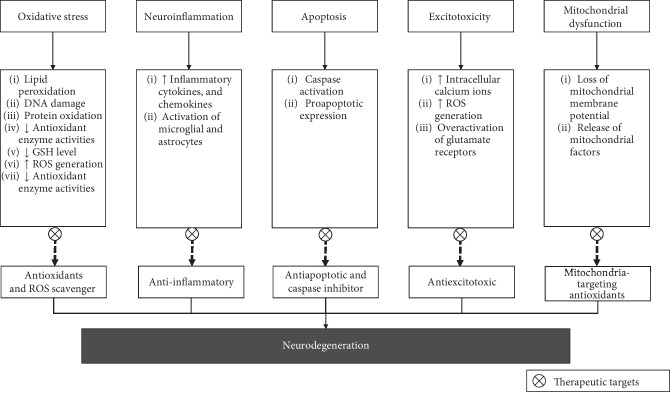
The potential therapeutic targets on various mechanisms of neurodegeneration.

Since the multifactorial pathological mechanisms are associated with neurodegeneration, targeting multiple mechanisms of actions is a promising strategy for the prevention and therapeutic of neurodegenerative diseases. Several potential therapeutic targets to combat neurodegenerative diseases could be explored (see Figure 2).

## 3. Neuroprotective Activities of Natural Products and Their Bioactive Compounds

The therapeutic potential of natural products and their bioactive compounds to exert a neuroprotective effect on the pathologies of neurodegenerative diseases will be discussed in more detail in this section.

### 3.1. Honey

Honey, a beehive product, is the natural sweet substance with therapeutic and nutritional values. It is highly rich in bioactive compounds and antioxidants such as polyphenols. It is widely used as a nutrient supplementation and in traditional medicine. Since the last two decades, honey has been explored for its gastroprotective, hepatoprotective, reproductive, hypoglycemic, antioxidant, antihypertensive, antibacterial, antifungal, anti-inflammatory, immunomodulatory, wound healing, cardioprotective, and antitumor effects [33–40]. In general, pure honey contains over 200 compounds, consisting mainly of carbohydrates (monosaccharides: glucose and fructose; disaccharides: sucrose and maltose), protein (amino acids and enzymes), minerals, vitamins (vitamin B6, riboflavin, niacin, and thiamine), phenolic compounds (flavonoids and phenolic acids), and volatile substance (responsible for the characteristic aroma of honey) [41–46].

In kainic acid-induced excitotoxicity animal model, pretreatment with honey significantly attenuated oxidative stress, neuroinflammation, and apoptosis in the cerebral cortex, cerebellum, and brainstem as well as progression of neuronal damage in the piriform cortex of kainic acid-induced rats [47–49]. Hence, honey confers its neuroprotection against the deleterious effect of kainic acid through its antioxidants and anti-inflammatory and antiapoptotic properties, thereby protecting brain from neuronal loss and neurodegeneration. In addition, honey has been reported to reduce brain oxidative stress and improve morphological impairment of the hippocampus and medial prefrontal cortex in stressed rats [50–55]. Honey also attenuates the cognitive impairment caused by chronic cerebral hypoperfusion-induced neurodegeneration [56] and ameliorates oxidative stress in the rat midbrain against repeated paraquat exposure [57]. Meanwhile, in normal rats, consumption of honey could prevent morphological impairment of the hippocampus and improve the spatial memory performance of adult male rats [58]. These findings were clinically supported by a study done in healthy postmenopausal women [59] reporting the improvement in total learning and immediate memory observed in healthy postmenopausal women after receiving the supplementation of honey for 16 weeks. Among the compounds found in these honeys are phenolic acids (gallic, syringic, coumaric, benzoic, cinnamic, caffeic, ferulic, and chlorogenic acids), flavonoids (catechin, naringenin, kaempferol, pinobanksin-3-O-propionate, pinobanksin-3-O-butyrate, and quercetin), minerals (potassium, calcium, sodium, and magnesium), amino acids (aspartic acid, serine, glutamic acid, glycine, threonine, alanine, proline, tyrosine, valine, methionine, lysine, isoleucine, and leucine), and vitamins (vitamin B3 and vitamin C) [43, 60–62]. Hence, the neuroprotective effects of honey are likely to be exerted at different stages of neurodegeneration and play prominent roles in early events. Therefore, honey could be considered as a potential candidate in mitigating the oxidative stress, inflammation, and apoptosis in neurodegenerative diseases as well as improving spatial memory performance and attenuating the cognitive impairment.

These protective effects of honey could be resulting from the presence of flavonoids and phenolic acids as well as other bioactive compounds in honey. Furthermore, the synergistic effect of these bioactive compounds present in honey could also contribute to the neuroprotection action of honey against neurodegeneration. Several important constituents including polyphenols found in honey have been suggested to play the neuroprotective role by reduction of oxidative stress, attenuation of neuroinflammation, the improvement of memory, learning and cognitive function, and protection against neuronal injury and neurodegeneration [63–66].

### 3.2. Propolis

Another bee product, propolis is a complex resinous mixture produced by bees and is used for defense against intruders and reconstruction of beehive. Propolis is mainly composed of resins, balms, wax, essential oils, pollen, amino acids, micronutrients, and vitamins and other organic compounds [67–70]. These organic compounds found in the propolis include phenolic compounds, flavonoids, terpenes, aromatic aldehydes, and alcohols [71]. The properties and chemical composition of propolis differ depending on various factors including the geographical origin, the season and time of collection, and types of plant sources [67, 68]. Propolis possesses numerous biological and pharmacological properties, such as antifungal, anticancer, antidiabetic, antibacterial, anti-inflammatory, and antioxidant activities [67, 68, 70, 72, 73]. These beneficial effects of propolis could have been attributed to its bioactive compounds as well as the combined actions of these compounds [69].

Propolis has been reported to attenuate nitric oxide level, glutamine synthetase activity, oxidative stress, tumor necrosis factor-alpha (TNF-*α*), and caspase-3 activity, reduce seizures, and prevent neuronal loss in kainic acid-induced excitotoxicity rat model [74–76]. These findings suggested that propolis exhibits its inhibitory effect on the oxidative stress, proinflammatory cytokines, and apoptosis as well as protection against neuronal damage through its anti-inflammatory, antioxidant, and antiapoptotic properties. The beneficial action of propolis might be due to the presence of phenolic compounds and flavonoids [76].

Moreover, Ni et al.'s study [77] has reported that Brazilian green propolis reduced the hydrogen peroxide-induced mitochondria-derived ROS generation and nuclear DNA oxidative damage marker, 8-oxo-2′-deoxyquanosine in human neuroblastoma (SH-SY5Y) cell line. This indicated that the propolis could attenuate the oxidative stress in neuronal cells. In addition, propolis also showed to reverse the interleukin-1 beta- (IL-1*β*-) induced and fibrillar A*β*-induced impairment of brain-derived neurotrophic factor- (BDNF-) induced activity-regulated cytoskeleton-associated protein (Arc) mRNA and protein expressions, upregulate the mRNA expression of BDNF through phosphoinositide 3-kinase- (PI3K-) dependent pathways, and increase the expression of critical factors of synapse efficacy. Hence, this study indicated that propolis may have the ability to prevent neurodegenerative damaged synapse efficacy, which could relate to cognitive impairment in neurodegenerative diseases, through antioxidant property. This finding supported the previous studies [78, 79] that reported the neuroprotective effect of Brazilian green propolis and its main components (caffeoylquinic acid derivatives, artepillin C, and p-coumaric acid) against retinal damage *in vitro* [78] and against oxygen-glucose deprivation/reoxygenation-induced neuronal damage [79]. The Brazilian green propolis displays its neuroprotective effect through its antioxidant mechanism of actions, which could be attributed to the synergistic effect of its main components (caffeoylquinic acid derivates, artepillin C, and p-coumaric acid).

Moreover, Ueda et al. work [80] reported that ethanol extract of Brazilian green propolis protected N2a cells against amyotrophic lateral sclerosis-associated mutant SOD1^G85R^-mediated neurotoxicity and reduced intracellular aggregates of mutant SOD1^G85R^ by autophagy induction. This neuroprotective effect could be due to the role of its active ingredients, like flavonols, against mutant SOD1^G85R^-induced neurotoxicity. In addition, Ueda et al. [80] also reported that its active ingredients, such as kaempferide and kaempferol, also prevented mutant SOD1^G85R^-induced toxicity and reduced aggregated mutant SOD1^G85R^ as well as suppressed mutant SOD1^G85R^-induced superoxide in mitochondria. This study suggested that both kaempferide and kaempferol exert an antioxidant activity which involved in the neuroprotection against mutant SOD1^G85R^-induced toxicity. However, only kaempferol could reduce intracellular aggregate and induced autophagy through adenosine monophosphate- (AMP-) activated protein kinase (AMPK)—the mammalian target of rapamycin (mTOR) pathway. Subsequently, kaempferol inhibited mutant SOD1^G85R^-induced toxicity. These results suggested that ethanol extract of Brazilian green propolis as well as kaempferol may have a potential to be neuroprotective agents.

Another study done by Nanaware et al. [81] on the macerated ethanolic extract of Indian propolis in an animal model of Alzheimer's disease reported that treatment with propolis could reverse the cognitive impairment, inhibit acetylcholinesterase, and increase brain monoamine level as well as improve memory deficits by increasing plasma BDNF level in A*β* (25–35)-induced rats. From this study, it indicated there could be multiple mechanisms of actions participated in the neuroprotection of propolis against A*β* (25–35)-induced neurodegeneration.

In the cerebral ischemia-induced oxidative injury stroke mouse model, water-extracted brown propolis was reported to restore the endogenous enzymatic antioxidant activity and subsequently reduce lipid peroxidation and infarct volume [82]. The treatment with water-extracted brown propolis also improved sensory-motor impairment and neurological deficits [82]. These findings indicated that propolis confers its neuroprotective effect against neuronal oxidative damage following stroke via its antioxidant mechanism which is mediated by the endogenous antioxidant enzyme system.

Taken together, all these findings suggest that propolis displays neuroprotective through multiple pathways which might be attributed by the presence of bioactive compounds in propolis as well as their combination of multitarget compound acting on the different pathways. Several studies have reported on the neuroprotective effect of compounds present in the propolis in various study models of neurodegenerative diseases; for example, kaempferol [80, 83], chrysin [84], pinocembrin [85–96], and caffeic acid phenethyl ester [97–108]. These studies reported and suggested that these bioactive compounds may protect against neurodegenerative characteristics of neurodegenerative diseases and the multiple mechanisms of the neuroprotective effect of compounds tested could be potentially involved. Those suggested mechanisms included counteracting oxidative damage, modulating events associated with apoptosis, inhibiting glial activation, and suppressing the production of proinflammatory mediators as well as improving the cognitive impairment and motor performances.

### 3.3. *Withania somnifera*

*Withania somnifera*, commonly known as Indian ginseng or Ashwagandha, is an important medicinal plant being used in Ayurveda and belongs to family Solanaceae. It is a perennial herb covered with hairs. The characterization of phytochemicals has revealed the presence of different chemical constituents extracted and isolated from the different parts of *Withania somnifera* plant, which include steroidal lactones, alkaloids, flavonoids, tannin, withanolides, and several sitoindosides [109].

In an animal model of Parkinson's disease, study has shown that pretreatment with ethanolic crude extract of *Withania somnifera* reduced oxidative stress and increased catecholamine content in 6-hydroxydopamine- (6-OHDA-) induced rats [110]. In another animal model of Parkinson's disease, administration of *Withania somnifera* root extract improved behavioral alternations of mice in the rotarod test, hang test, and stride length measurement, restored the antioxidant status, and reduced lipid peroxidation as well as increase striatal catecholamine contents in 1-methyl-4-phenyl-1,2,3,6-tetrahydropyridine- (MPTP-) induced mice [111, 112]. In a rotenone-induced mouse model of Parkinson's disease, *Withania somnifera* extract restored the reduced glutathione (GSH) level and activity level of antioxidant enzymes, reduced lipid peroxidation, and normalized nitric oxide levels [113]. The extract of *Withania somnifera* also attenuated the increase of acetylcholinesterase activity and restored dopamine level and mitochondrial electron transport chain enzyme complex activities [113]. Hence, these findings suggested that *Withania somnifera* extracts reduced oxidative stress and mitochondrial dysfunction and the beneficial role of *Withania somnifera* extracts seems to be primarily based on its antioxidant property.

In 3-nitropropionic acid-induced Huntington's like mouse model, administration of *Withania somnifera* root extracts decreased lipid peroxidation and levels of nitrite and lactate dehydrogenase and restored the antioxidant enzymes levels and adenosine triphosphate (ATP) synthesis as well as attenuated mitochondrial enzyme complex activities in the striatum and cortex of 3-nitropropionic acid-induced rats [114]. This indicated that *Withania somnifera* root extracts could reduce oxidative stress and mitochondrial dysfunction. Besides, *Withania somnifera* root extracts improved motor function assessed by locomotor and rotarod tests and improved muscle activity as observed in the rotarod and limb withdraw test [114]. This finding suggested that the neuroprotective action of *Withania somnifera* could be partly attributed due to its free radical scavenging and antioxidant activities and its ability to decrease the loss of ATP from mitochondria as well as beneficial effect on improving motor performance.

In amyotrophic lateral sclerosis-associated mutant SOD1^G93A^-induced mice, *Withania somnifera* root extract ameliorated the motor performance and delayed the disease progression rate [115]. Besides, the extract reduced misfolded SOD1 species levels and upregulated heat-shock protein in the spinal cord of SOD1^G93A^-induced mice [115]. This finding suggested that *Withania somnifera* root extracts could inhibit SOD1 misfolding through induction of molecular chaperons and reduction of oxidative stress. The extract also attenuated glial activation (glial fibrillary acidic protein (GFAP) and ionized calcium-binding adaptor molecule-1 (Iba-1)) and NF-*κ*B phosphorylation in the spinal cord of SOD1^G93A^-induced mice and promoted autophagy [115]. This indicated that the extract could modulate inflammation to minimize neuronal damage through the inhibition of NF-*κ*B activation and thereby induced the activity of autophagy.

In a clinical application, *Withania somnifera* has shown promising results in a randomized, double-blind, placebo-controlled clinical pilot study in enhancing immediate and general memory and improving cognitive function in adult with mild cognitive impairment [116]. Another randomized, double-blind, placebo-controlled adjunctive study reported that *Withania somnifera* extract could ameliorate cognitive dysfunction in adults with bipolar disorder [117]. Other than that, a clinical study has suggested that *Withania somnifera* could improve cognitive and psychomotor performance in healthy human participants [118].

Collectively, the possible mechanism underlying the neuroprotective actions of *Withania somnifera* could be mediated through several pathways. Several studies have suggested that the protective effect of withanoside IV [119], withanone [120–122], withaferin A [123–125], and withanolide A [126–130] and other compounds as well as the synergistic action of multiple compounds present in *Withania somnifera* extract could contribute towards beneficial effect of *Withania somnifera* in the pathogenesis of neurodegenerative diseases.

### 3.4. Ginseng

Ginseng is a perennial herb belonging to the *Panax* genus of the Araliaceae family. It has been used as a traditional herbal medicine, especially in China, Korea, and Japan. The most frequently studied ginseng is *Panax ginseng* C. A. Meyer (Asian ginseng or Korean ginseng) [131]. Ginseng and its chemical constituents have exhibited various beneficial effects, which include antioxidant, anti-inflammatory, antiapoptotic, anticancer, antifatigue, antidiabetic, and antiaging [132–139]. Apart from that, ginseng and its constituents are also known to have a beneficial effect on the central nervous system [140–143]. The major active constituents of ginseng are ginsenosides, which are derivatives of the triterpenoid dammarane. The most extensively investigated ginsenosides are ginsenoside Rb1, Rd, Re, and Rg1 [144, 145].

In an animal model of Parkinson's disease, Korean red ginseng could improve the behavioral impairment of mice in the pole test, inhibit dopaminergic neuronal death, and decrease cyclin-dependent kinase 5 (Cdk5) and p25 expressions as well as increase p35 expression in the substantia nigra and striatum of MPTP-induced mice [146]. Further study was done by the same group reporting that Korean red ginseng restored the MPTP-induced proteomic changes in the striatum [147]. These changes were related to the energy metabolism, oxidative phosphorylation, and neurodegenerative diseases. A separate study done by the same group reported that Korean red ginseng also alleviated the protein expression profile in the substantia nigra that involved in the neuronal development and energy metabolism for the neuronal survival as well as neuroprotection [148]. Meanwhile, in the cellular model, Korean red ginseng treatment inhibited 1-methyl-4-phenylpyridinium ion- (MPP^+^-) induced apoptosis on rat pheochromocytoma (PC12) cells by preventing the decrease of cell viability and apoptosis and decreasing mRNA expressions of caspase-3 and caspase-9 [149]. Furthermore, this group also reported that Korean red ginseng treatment alleviated behavioral dysfunction induced by MPTP and enhanced differentiation and proliferation of endogenous neural stem cells in the subventricular zone of MPTP-induced mice [150].

Another research group [151] has reported that administration of Korean red ginseng attenuated MPTP-induced locomotor impairment assessed in the pole test, rotarod test, and nest-building behavior test which was correlated to the reduction of MPTP-induced nigrostriatal dopaminergic neuronal damage [151]. In addition, Korean red ginseng extracts attenuated apoptosis, inhibited microglia and astrocyte activation, and suppressed proinflammatory modulator expressions in the substantia nigra and striatum after MPTP induction. Pretreatment with Korean red ginseng also decreased phosphorylation of extracellular signal-regulated kinase (ERK), Jun-N-terminal kinase (JNK), and p38 protein as well as inhibited blood-brain barrier disruption [151]. Moreover, Korean red ginseng extract increased nuclear factor erythroid 2-related factor 2 (Nrf2) protein expression and consequently increased mRNA expressions of enzymes heme oxygenase-1 (HO-1), nicotinamide adenine dinucleotide phosphate hydrogen (NADPH) quinone oxidoreductase-1 (NQO1), and gamma-glutamate cysteine ligase regulatory subunit (GLCs) [151]. These findings suggested the Korean red ginseng could reduce motor deficits and protect dopaminergic neurons against MPTP-induced neurotoxicity possibly through antiapoptotic, antioxidant effect by the activation of Nfr2 pathways and anti-inflammation by inhibition of mitogen-activated protein kinases (MAPKs) and nuclear factor kappa-light-chain-enhancer of activated B cells (NF-*κ*B) pathways and maintaining the integrity of blood-brain barrier as well as suppression of overexpression of Cdk5 and cleavage of p35 to p25 in the nigrostriatal system of MPTP-induced mice.

The study done by Hu et al. [152] reported that water extract of *Panax ginseng* C. A. Meyer protected neuronal cell, prevented the cellular morphological deterioration, reduced DNA fragmentation and percentage of apoptotic cells as well as attenuated the induction of Bax/Bcl-2 ratio, cytosolic cytochrome-c, and cleaved caspase-3 in MPP^+^-induced cytotoxicity in SH-SY5Y cells, indicating the protective effect of ginseng extract against apoptosis. The extract also inhibited the accumulation of intracellular Ca^2+^ and the production of intracellular ROS generation in MPP^+^-induced cytotoxicity in SH-SY5Y cells [152], suggesting the suppression of oxidative stress by the ginseng extract. These findings suggested that water extract of P*anax ginseng* C. A. Meyer exerts its protective effect against MPP^+^-induced apoptosis and cytotoxicity possibly through its antioxidant property and antiapoptotic activity by suppressing ROS generation and oxidative stress and inhibiting mitochondria-dependent apoptotic pathway [152].

In an animal model of Huntington's disease, pretreatment with Korean red ginseng extract improved neurological impairment and striatal neuronal cell loss in 3-nitropropionic acid-induced Huntington's-like mice [153]. Furthermore, Korean red ginseng extract also inhibited microglial activation (Iba-1 immunoreactivity and mRNA expression of OX-42) and proinflammatory mediators (iNOS, IL-1*β*, IL-6, and TNF-*α*) mRNA expressions as well as increased the activation of p38, JNK, ERK MAPKs, and NF-*κ*B signaling pathways in striatum of 3-nitropropionic acid-induced Huntington's-like mice [153]. This finding suggested that Korean red ginseng attenuated 3-nitropropionic acid-induced striatal neurotoxicity possible through anti-inflammatory activity by inhibiting activation of MAPKs and NF-*κ*B signaling pathways and activation of microglial as well as expression of proinflammatory mediators in the striatum.

In an acute autoimmune encephalomyelitis animal model of multiple sclerosis, pretreatment with Korean red ginseng mitigated spinal demyelination, reduced the microglial Iba-1 immunoreactivity of white matter, and suppressed the mRNA expressions of the inflammatory mediators and neurotoxic factors in the spinal cord of experimental autoimmune encephalomyelitis-induced rats [154]. Pretreatment with Korean red ginseng extract also reduced the protein expression levels of p-p38 MAPK and NF-*κ*B/p65 [154]. Hence, this indicated that Korean red ginseng extracts alleviated demyelination in the spinal cord of experimental autoimmune encephalomyelitis-induced rats by inhibiting the phosphorylation of p38 MAPK and NF-*κ*B signaling pathways.

In rat model of Alzheimer's disease, extract of *Panax ginseng* has been reported to enhance learning and memory ability, reduce oxidative damage, and inhibit the receptors for advanced glycation end product (RAGE) and NF-*κ*B expressions in the cortex and hippocampus of advanced glycation end product- (AGE-) induced rats [155]. According to a study on fermented ginseng [156], treatment with fermented *Panax ginseng* extract ameliorated memory impairment in scopolamine-induced amnesia ICR mice as well as in transgenic mice and reduced accumulation of A*β*42 protein in the brain of transgenic mice. This indicated that *Panax ginseng* improved cognitive impairment and mitigated Alzheimer's disease-like pathophysiological changes through downregulating RAGE and thus inhibiting NF-*κ*B activation.

Taken together, these findings indicated that ginseng exhibits its neuroprotective properties and improves cognitive and motor functions through various mechanisms. This could be due to the presence of bioactive compound ginsenosides in ginseng. Several studies have reported on the neuroprotective effect of ginsenosides, particularly ginsenosides Rg1, in various neurodegeneration models [157–169]. The neuroprotective effects of these compounds may have been mediated by antiapoptotic, antioxidant, and anti-inflammatory properties, modulating events associated with apoptosis, inhibiting mitochondrial dysfunction well as improving the cognitive impairment and motor performances.

In the clinical application, the clinical efficacy of *Panax ginseng* was reported to enhance the cognitive performances in patients with Alzheimer's disease in an open-label study [170]. For the bioactive compounds in ginseng, the pharmacokinetics and safety profile of ginsenoside Rd has been evaluated and reported to have a favorable pharmacokinetics and safety profile in healthy adults [171]. Moreover, the efficacy and safety of ginsenoside Rd for acute ischemic stroke were evaluated in randomized, double-blind, placebo-controlled, phase II, multicenter trial study [172]. They reported that ginsenoside Rd may have some potential benefit, partly improved neurological deficits in patients with acute ischemic stroke [172]. Further study on the effectiveness of ginsenoside Rd for acute ischemic stroke was performed and the study reported ginsenoside Rd improved the primary outcomes of acute ischemic stroke and did not promote severe adverse event profile [173].

### 3.5. *Uncaria rhynchophylla*

*Uncaria rhynchophylla*, which belongs to family Rubiaceae, is a medicinal herb used in the traditional Chinese medicine. Active components found in the extract of *Uncaria rhynchophylla* are the alkaloids, which are rhynchophylline, isorhynchophylline, hirsutine, hirsuteine, corynanthine, corynoxine, and dihydrocorynantheine [174, 175]. Among these alkaloids, rhynchophylline and isorhynchophylline are the most widely studied and have been known as neuroprotective compounds [176].

In kainic acid-induced excitotoxicity rat model, *Uncaria rhynchophylla* extract exhibited free radical scavenging activity and suppressed lipid peroxidation [177, 178]. *Uncaria rhynchophylla* also has been reported to exhibit protection against kainic acid-induced neuronal damage, the reduction of microglial activation, neuronal nitric oxide synthase (nNOS), iNOS, and apoptosis [179], and the attenuation of GFAP and S100 calcium-binding protein B (S100B) expressions [180, 181] in the hippocampal region. Pretreatment with *Uncaria rhynchophylla* extract before kainic acid administration also has increased the survival of neurons and reduced the epileptiform discharges in the hippocampus [180]. In the experimental model of global ischemia, *Uncaria rhynchophylla* methanol extract inhibited COX-2 mRNA and protein levels in the hippocampus of rat and inhibited TNF-*α* and nitric oxide levels in BV-2 microglial cell [182]. Moreover, *Uncaria rhynchophylla* inhibited the formation of A*β* fibrils and also dissembled preformed A*β* fibrils in A*β* (1–40)- and A*β* (1–42)-induced toxicity of Alzheimer's disease model [183].

The neuroprotective effect of *Uncaria rhynchophylla* has been also reported in Parkinson's disease experimental model. Shim et al. [184] reported that *Uncaria rhynchophylla* reduced neuronal cell death and ROS generation, restored the GSH level, and prevented the caspase-3 activity in 6-OHDA-induced toxicity in PC12 cells as well as reduced neuronal loss in dopaminergic neurons in the substantia nigra in 6-OHDA-induced rats. This finding was supported by the study done by Pal and Kumar [185] that demonstrated that *Uncaria rhynchophylla* exhibited anti-Parkinson's activity in 6-OHDA rat model. In another model of Parkinson's disease, *Uncaria rhynchophylla* has been reported to increase cell viability, attenuate dopaminergic neuronal loss of substantia nigra and striatum, inhibit heat-shock protein 90 and apoptosis, and thereby induce autophagy through MAPKs and PI3K-serine/threonine protein kinase (Akt) pathways in MPP^+^-induced SH-SY5Y cells and MPTP-induced mouse model [186].

Taken together, all these findings suggest that *Uncaria rhynchophylla* display neuroprotective action in protecting neuronal damage through multiple pathways which could be due to the beneficial effect of active compounds as well as the combination effect of those compounds present in *Uncaria rhynchophylla*. Several studies have reported on the neuroprotective effect of alkaloid in *Uncaria rhynchophylla* such as hirsutine [187], rhynchophylline [188–191], and isorhynchophylline [192–195]. These compounds exert antioxidant effect by reducing ROS generation and enhancing the antioxidant defense system, anti-inflammatory effect by inhibiting the production of inflammatory mediators and antiapoptotic effect by modulating the event associated with apoptosis and inhibiting caspase activation, and behavioral effect on cognitive functions.

### 3.6. Marine Macroalgae (Seaweeds)

Marine macroalgae, commonly known as seaweeds, are plant-like organisms that generally live in coastal areas. They can be divided into three groups based on their colors, which are brown algae (Phaeophyceae), red algae (Rhodophyceae), and green algae (Chlorophyceae) [196]. Their colors are associated with the presence of different phytopigments in algae: chlorophyll is for green algae, phycobilin is for red algae, and fucoxanthin for brown algae [197]. They contain diverse bioactive compounds which include phenolic compounds, protein, peptides, amino acids, pigments, and phenols [198]. Several studies have reported the health beneficial effect of algae as well as the bioactive compounds of different algae [196, 198, 199].

*Eisenia bicyclis* (Kjellman) Setchell, a perennial brown alga, belongs to the Laminariaceae family. This species contains several bioactive compounds, including phlorotannins, polysaccharides, pyropheophytin, sterol, lipids, tripeptides, and oxylipins. Ahn et al.'s study [200] has investigated the neuroprotective effect of *Eisenia bicyclis* methanol extract and its soluble fractions together with the isolated phlorotannins on A*β* (25–35)-induced cytotoxicity in PC12 cells. In this study, six phlorotannins were isolated from ethyl acetate fraction of *Eisenia bicyclis* methanol extract which were phloroglucinol, dioxinodehydroeckol, eckol, phlorofucofuroeckol A, dieckol, and 7-phloroeckol [200]. The study also reported that the neuroprotective activity of *Eisenia bicyclis* methanol extract and its subfractions ethyl acetate and *n*-butanol fractions together with the isolated phlorotannins from ethyl acetate fraction was due to their ability to reduce intracellular ROS production in A*β* (25–35)-induced cytotoxicity in PC12 cells [200]. In addition, the isolated phlorotannins eckol, phlorofucofuroeckol A, and 7-phloroeckol exert their neuroprotective effect through suppression of intracellular ROS production and restoring intracellular Ca^2+^ level in A*β* (25–35)-induced cytotoxicity in PC12 cells [200]. This study clearly demonstrated that *Eisenia bicyclis* and its isolated phlorotannins attenuated oxidative damage and neuronal cell death in A*β* (25–35)-induced cytotoxicity in PC12 cells.

Silva et al. [201] have investigated the neuroprotective effect of several seaweeds in 6-OHDA-induced toxicity in SH-SY5Y cells which included *Padina pavonica*, *Sargassum muticum*, *Saccorhiza polyschides*, *Codium tomentosum*, and *Ulva compressa*. The study reported that seaweed extracts had increased the cell viability, reduced oxidative stress, protected mitochondrial membrane potential, and reduced caspase-3 activity. This finding indicated that these seaweeds exert their neuroprotective effects through antioxidant activity and antiapoptotic property. Silva et al. [201] suggested that this neuroprotection action could be mediated by the presence of antioxidant compounds in the seaweed extracts. The same research group has also reported the neuroprotective effect of several fractions obtained from brown seaweed, *Bifurcaria bifurcata* (belongs to Sargassaceae family) against 6-OHDA-induced toxicity in SH-SY5Y cells [202]. Fraction F4 obtained from *Bifurcaria bifurcata* was reported to exhibit the best neuroprotective effect by protecting mitochondrial membrane potential, reducing hydrogen peroxide production, and inhibiting apoptosis through caspase-3 activity. The authors suggested that this effect could be attributed by the presence of bioactive compounds like eleganolone and eleganonal, which could act on multiple targets through different mechanisms of actions.

Another brown seaweed, *Ishige foliacea* belongs to the Ishigeaceae family and has known to contain polyphenolic compound known as phlorotannin. Heo et al.'s study [203] has reported that a specific phlorotannin, diphlorethohydroxycarmalol, isolated from *Ishige foliacea*, increased cell viability and prevented cells from damage induced by hydrogen peroxide in murine hippocampal neuronal cells (HT22). Diphlorethohydroxycarmalol also reported to inhibit caspase-3, caspase-9, and poly (ADP-ribose) polymerase (PARP) cleavage and decrease ROS generation as well as inhibit lipid peroxidation and intracellular Ca^2+^ levels [203]. Another study done by Um et al. [204] demonstrated the effect of phlorotannin-rich fraction from *Ishige foliacea* in scopolamine-induced memory impairment in mice model. The study reported that it improved memory impairment in mice, reduced brain acetylcholinesterase activity, upregulated the protein expression level of BDNF and tropomyosin-related kinase receptor type B (TrkB), and suppressed oxidative stress by decreasing lipid peroxidation level and increasing the GSH level and superoxide dismutase activity as well as increased the phosphorylation levels of cyclic AMP responsive element binding protein (CREB) and ERK in the cerebral cortex and hippocampus [204]. All these above findings suggested that marine algae display neuroprotective action in protecting neuronal damage and improving memory impairment through multiple pathways which might be attributed due to their bioactive compounds as well as the synergistic effect of these compounds.

Recently, sulfated polysaccharides isolated from marine algae have attracted the interest of scientists for their potential use in pharmaceutical application, mainly in the design of novel drug delivery system [205–208]. Various studies have reported the biological activities of sulfated polysaccharides. Major sulfated polysaccharides found in marine algae include fucoidan from brown seaweed, ulvan from green seaweed, and carrageenan from red seaweed. Several studies have reported on the neuroprotective effect of fucoidan in various neurodegeneration models [209–214]. Luo et al.'s study [209] demonstrated that fucoidan, isolated from *Laminaria japonica*, attenuated MPTP-induced locomotor activity deficits, prevented depletion of striatal dopamine and its metabolite, dihydroxyphenylacetic acid (DOPAC) level, reduced loss of dopaminergic neurons and tyrosine hydroxylase protein expression, and attenuated the increase of lipid peroxidation level and decreases of antioxidant enzyme activities as well as total antioxidant capacity in the substantia nigra pars compacta of MPTP-induced mice. Fucoidan also protected against MPP^+^-induced neurotoxicity in MN9D cells [209]. In the 6-OHDA rat model of Parkinson's disease, fucoidan from *Laminaria japonica* also reduced the dopaminergic neuronal loss in the substantia nigra pars compacta and dopaminergic fibers, inhibited the increase of NADPH oxygenase-1 (NOX1), oxidative stress, and microglial activation in the substantia nigra pars compacta, and attenuated motor dysfunction in 6-ODHA-induced rats [210]. In another rat model of Parkinson's disease, fucoidan from *Laminaria japonica* alleviated Parkinson's disease-like behaviors, reduced nigral dopaminergic neuronal loss and oxidative stress, prevented the reduction on dopamine and its metabolites, and enhanced mitochondrial respiratory function through the peroxisome proliferator-activated receptor-gamma coactivator-1*α* (PGC-1*α*)/Nrf2 pathway in rotenone-induced rats [211].

Another fucoidan, isolated from *Fucus vesiculosus*, inhibited the disruption of mitochondrial membrane potential and attenuated lipid peroxidation and the decrease of glutathione/glutathione disulfide ration as well as the activities of caspase-3/caspase-7 in 6-OHDA-induced neurotoxicity in SH-SY5Y cells [214]. Meanwhile, fucoidan from *Undaria pinnatifida* improved cell viability, prevented apoptosis through inhibition of caspase-3 activation, and enhanced the antioxidant dense system through superoxide dismutase activity and GSH content, in A*β* (25–35)- and D-galactose-induced neurotoxicity in PC12 cells [212]. This fucoidan from *Undaria pinnatifida* also regulated cholinergic system, ameliorated spatial learning and memory impairment, improved antioxidant capacity, and decreased A*β* deposition, as well as maintained the density and shape of hippocampus CA1 neurons in D-galactose-induced mice [212]. These findings were supported by the recent study done by Alghazwi et al. [213] that demonstrated the neuroprotective activities of fucoidans from *Fucus vesiculosus* and *Undaria pinnatifida* against A*β* (1–42)-induced and hydrogen peroxide-induced cytotoxicity in PC12 cells. These fucoidans decreased A*β* (1–42) aggregation, decreased A*β* (1–42) aggregation, reduced A*β* (1–42)-induced and hydrogen peroxide-induced cytotoxicity in PC12 cells, and reduced A*β* (1–42)-induced apoptosis and enhanced neurite outgrowth activity [213].

### 3.7. Cyanobacteria

Cyanobacteria, commonly known as blue-green alga, are prokaryotic, photosynthetic, autotrophic organisms closely related to bacteria. They have gained many attentions from researchers for its possible pharmacological properties and therapeutic effects on various medical conditions [199, 215–218]. *Spirulina platensis*, a cyanobacterium belonging to the Oscillatoriaceae family, is a planktonic multicellular filamentous, alkaliphilic cyanobacterium. It has been widely studied and known to have abundant nutritive elements. *Spirulina platensis* contains protein, polyunsaturated fatty acids, carotenoids, polysaccharides, vitamins, and minerals [219]. In a study [220], a polysaccharide derived from *Spirulina platensis* could attenuate the reduction in the immunoreactivity and mRNA expressions of tyrosine hydroxylase and dopamine transporter in the substantia nigra and attenuate the decrease in the dopamine levels and the increase in dopamine metabolism rate as well as increase the antioxidant enzyme activities such as superoxide dismutase and glutathione peroxidase in the serum and midbrain of mice with MPTP-induced Parkinson's disease. Subsequently, it could protect against MPTP-induced dopaminergic neuronal loss in the substantia nigra. However, no significant change was observed on the monoamine oxidase B activity in the serum and midbrain of mice with MPTP-induced Parkinson's disease study [220]. This evidence indicated that polysaccharide derived from *Spirulina platensis* exhibited its neuroprotective effect on dopaminergic neurons and dopamine levels through antioxidant mechanism, but not through the inhibition of monoamine oxidase B.

In another study using 6-OHDA-induced Parkinson's disease rat model [221], *Spirulina platensis* attenuate the depletion of striatal dopamine and its metabolite and the increase of nitrite and lipid peroxidation levels as well as reduce the apomorphine-induced rotational behavior. In addition, the authors also reported that *Spirulina platensis* also blocked the reduction of immunoreactivity of tyrosine hydroxylase and dopamine transporter and reduced the increase of the immunoreactivity of iNOS and COX-2 in the striatal of 6-OHDA-induced rats [221]. From this study, it indicated that *Spirulina platensis* presented potent anti-inflammatory and antioxidant activities which contribute to its neuroprotective actions against 6-OHDA-induced Parkinson's disease.

Bermejo-Bescós et al.'s study [222] has reported that *Spirulina platensis* protean extract and the biliprotein phycocyanin isolated from the extract attenuate the decrease in cell survival and the increase of oxidative stress in SH-SY5Y cells against iron-induced toxicity. The same study also indicated that *Spirulina platensis* protean extract and phycocyanin exhibited its protection against iron-induced toxicity by increasing the GSH level and the enzymes activities in the glutathione metabolism pathway and decreasing the lipid peroxidation level as well as scavenging ROS [222]. Thus, this finding suggested that *Spirulina platensis* protean extract, a potent antioxidant which acts through a mechanism related to its antioxidant activity, has the chelating capability and free radical scavenging property, thereby protecting cells against radical-mediated cell death. This protective effect could be also contributed by the presence of the biliprotein C-phycocyanin [222, 223]. The protective effect of *Spirulina platensis* and C-phycocyanin was further expanded by the study [224] that demonstrated *Spirulina platensis* water extract and its active compound C-phycocyanin reduced cytotoxicity and inhibited the inflammation-related gene expressions (COX-2, TNF-*α*, IL-6, and iNOS) of BV-12 microglial cells induced by lipopolysaccharides.

Another cyanobacterium, *Spirulina maxima*, belonging to the Oscillatoriaceae family, has been known to have many physiologically active substances such as carotenoids, chlorophylls, polysaccharides, vitamins, and C-phycocyanin [225, 226]. In mouse model of Alzheimer's disease induced by A*β* (1–42), *Spirulina maxima* extract ameliorated learning and memory impairments in A*β* (1–42)-induced mice (assessed using the passive avoidance and Morris water maze tests), decreased the protein expression levels of hippocampal Aβ (1‐42), amyloid precursor protein (APP), and β-site APP cleaving enzyme 1 (BACE1) and attenuated the increase in acetylcholinesterase activity in the hippocampal oxidative stress of A*β* (1–42)-induced mice [227]. These findings indicated that *Spirulina maxima* extract ameliorated cognitive impairment through the inhibition of A*β* accumulation and acetylcholinesterase activity. *Spirulina maxima* extract also suppressed hippocampal oxidative stress of A*β* (1–42)-induced mice through enhancing the antioxidant system in the glutathione metabolism pathway by increasing the GSH level and attenuating the decrease of glutathione peroxidase and glutathione reductase protein expression levels [227]. This suggested that the inhibition of A*β* accumulation and acetylcholinesterase activity by *Spirulina maxima* extract could contribute to the suppression of oxidative stress. Moreover, *Spirulina maxima* extract increased the BDNF level, upregulated the phosphorylated PI3K and the phosphorylated Akt, and suppressed the phosphorylated glycogen synthase kinase-3*β* (GSK3*β*) in the hippocampal oxidative stress of A*β* (1–42)-induced mice [227]. Therefore, these findings suggested that *Spirulina maxima* extract inhibited the phosphorylation of hippocampal glycogen synthase kinase-3*β* through the activation of BDNF/PI3K/Akt signaling pathways. This could contribute to the inhibition of APP processing by regulating BACE1 protein and thereby ameliorate the cognitive impairment in A*β* (1–42)-induced mice.

Another study had demonstrated that the extract of *Spirulina maxima* could also prevent A*β*-induced oxidative stress and cell death through suppression of PARP cleavage, elevation of GSH levels, and upregulation of antioxidant enzymes and increasing cell viability death as well as reducing cytotoxicity in A*β* (1–42)-induced toxicity in PC12 cells [228]. Furthermore, *Spirulina maxima* extract increased the expression of BDNF and decreased the expressions of APP and BACE1 [228]. The same study also reported the protective effect of C-phycocyanin and chlorophylls against A*β* (1–42)-induced oxidative stress and cell death in PC12 cells, reporting a similar result was observed as *Spirulina maxima* extract [228]. Therefore, this study indicated that *Spirulina maxima* extract confers its neuroprotection action against A*β*-induced oxidative stress and cell death in PC12 cells by promoting the activation of the BDNF pathway. This could be attributed due to the presence of active compounds such as C-phycocyanin and chlorophylls as well as the synergistic effect of all active compounds present in the extract.

In animal model of Parkinson's disease, *Spirulina maxima* partially enhanced the content of dopamine and blocked lipid peroxidation in the striatum in MPTP-induced mice [229]. In another study using 6-OHDA-induced Parkinson's disease rat model, administration of *Spirulina maxima* improved locomotor activity, decreased 6-OHDA-induced rotational behavior, ROS generation, and nitric oxide level, inhibited the lipid peroxidation and mitochondrial dysfunction in the rat striatum, and prevented the striatal dopamine depletion of 6-OHDA-induced rats [230]. In kainic acid-induced excitotoxicity animal model, *Spirulina maxima* also reduced lipid peroxidation and the damaging neurobehavioral effect of kainic acid by preventing the spatial memory damage assessed by Morris water maze test [231]. Other study has reported that *Spirulina maxima* extract ameliorated scopolamine-induced memory impairment in mice [232, 233]. These findings indicated that *Spirulina maxima* exerts its neuroprotective effect through antioxidant activity.

Collectively, these studies indicated that Cyanobacteria display neuroprotective action against neurodegeneration through multiple mechanisms of actions, mainly through antioxidant activity. This could be attributed due to the presence of active compounds such as phycocyanin, carotenoids, and chlorophylls as well as the synergistic effect of all active compounds present in Cyanobacteria.

### 3.8. Other Natural Products

There were many other natural products that have been investigated for their neuroprotection potentials against neurodegeneration. A summary of natural products and their bioactive compounds with different neuroprotective activities according to the treating disease is presented in Tables 1–4.

## 4. Challenges, Limitations, and Recommendations for Future Research

Natural products and their isolated natural compounds have potentials to be neuroprotective and therapeutic agents as well as valuable resource for drug discovery against various neurodegenerative diseases. However, despite their promising neuroprotective activities against neurodegenerative diseases in the preclinical setting, translation of promising preclinical neuroprotective research to clinical application has proven challenging as there are no positive results in human clinical trials of neurodegenerative diseases.

There are a few challenges and limitations about natural products and their isolated natural compounds that could affect their clinical efficacy, which are their low bioavailability and limited water solubility, their physicochemical instability, their rapid metabolism, and their ability to cross blood-brain barrier. Further explanation could be reviewed in these literature studies [282–289]. Several natural compounds, for example, tea catechins [288, 290], resveratrol [287], and curcumin [282, 291–293], have been reported to show low bioavailability and limited stability as these natural compounds are sensitive to the degradation or transformation to inactive derivates [294, 295]. Consequently, this will limit their effectiveness. Moreover, the presence of the blood-brain barrier limits the natural compounds to access to the brain and to reach the action site. Subsequently, this will constrain the distribution to the brain tissues and lead to the low bioavailability.

To overcome these issues, the use of nanotechnology and nanocarrier-based approaches in the delivery of natural products and their isolated compounds may help and improve the therapeutic responses and enhance their effectiveness [296–302]. The incorporation of nanoparticle in the delivery system can increase the bioavailability of natural products and their compounds. The most common types of nanoparticle used are polymeric nanoparticles, nanogels, solid lipid nanoparticle, crystal nanoparticle, liposomes, micelles, and complexes with dendrimers. There have been several studies reported on the use of nanoparticle with natural products and their compound, for example, epigallocatechin-3-gallate for the treatments of Alzheimer's disease [302], rosmarinic acid in the management of Huntington's disease [303], and curcumin for brain disease [304].

## 5. Conclusion

The neuroprotective potentials of natural products and natural bioactive compounds against neurodegenerative diseases were supported by various experimental studies. Natural products and their important bioactive compounds are necessary to prevent and treat various neurodegenerative diseases without harmful side effects. Since the multifactorial involved in the pathological process of neurodegeneration, multiple mechanisms of actions involved are important toward neuroprotection strategies for the prevention and therapeutic of neurodegenerative diseases. Natural products and their bioactive compounds with the multiple mechanisms of actions in exhibiting their neuroprotective effects are preferable. Furthermore, the factor of having the ability to cross blood-brain barrier also plays an important role in the neuroprotective action of natural products and their bioactive compounds. Therefore, the development of new approaches/strategies, such as the application of nanotechnology in the delivery of natural products and compounds, that help to promote the access of neuroprotective to the brain, is needed to boost more neuroprotection action of natural products and their bioactive compounds for the prevention and therapeutic of neurodegenerative diseases.

## Figures and Tables

**Table 1:** Natural products and their bioactive compounds with neuroprotective activities in treating Parkinson's disease.

Plant extracts/phytochemicals (plant source)/natural products/substances	Study model	Neuroprotective activities	References
Arctigenin extracted from *Fructus arctii*	Rotenone-induced rats	(i) Improved behavioral changes	[234]
		(ii) Decreased dopaminergic neuronal loss in the substantia nigra pars compacta	
		(iii) Decreased *α*-synuclein immunopositive	
		(iv) Increased GSH and activities of superoxide dismutase and glutathione peroxidase	
		(v) Decreased malondialdehyde level	
		(vi) Decreased inflammatory markers (TNF-*α*, IL-1*β*, IL-6, interferon-gamma (IFN-*γ*), and prostaglandins E2 level) in the substantia nigra pars compacta	
		(vii) Decreased NF-*κ*B and COX-2 expressions in the substantia nigra pars compacta	
		(viii) Reduced GFAP and Iba-1 expressions	

*Apium graveolens* L.	MPTP-induced mouse	(i) Ameliorated MPTP-induced behavioral impairment	[235]
		(ii) Attenuated oxidative stress	
		(iii) Decreased monoamine oxidase activity	
		(iv) Protected dopaminergic neurons	

*Agaricus blazei* extract	Rotenone-induced mouse	(i) Restored the rotenone-induced motor and nonmotor behavioral deficits	[236, 237]
		(ii) Attenuated oxidative stress by decreasing TBARS level and increasing GSH level and superoxide dismutase, catalase, and glutathione peroxidase activities	
		(iii) Attenuated neuroinflammation markers (TNF-*α*, IL-1*β*, IL-6, COX-2, GFAP, Iba-1, iNOS expressions) in the substantia nigra pars compacta	
		(iv) Decreased NF-*κ*B level in the substantia nigra pars compacta	
		(v) Increased BDNF expression in the substantia nigra pars compacta	
		(vi) Attenuated the decrease in tyrosine hydroxylase expression in the substantia nigra pars compacta	
		(vii) Attenuated the depletion of striatal dopamine level	

Dihydromyricetin (DHM) (a natural flavonoid extracted from *Ampelopsis grossedentata*)	MPTP-induced mouse	(i) Attenuated MPTP-induced mouse behavioral impairments and dopaminergic neuron loss	[238]
		(ii) Attenuated the MPTP-induced deficit in movement balance	
		(iii) Improved exploratory and locomotor activity	
		(iv) Attenuated the decrease in tyrosine hydroxylase and vesicular monoamine transporter 2 expression in the striatum and substantia nigra pars compacta	

Agaropentaose, agaro-oligosaccharide monomer which is hydrolysates of agarose isolated from red algae	6-ODHA-induced neurotoxicity in SH-SY5Y cells	(i) Reduced intracellular ROS level	[239]
		(ii) Inhibited loss of mitochondrial membrane potential	
		(iii) Inhibited the activation of NF-*κ*B	
		(iv) Enhanced the activities of superoxide dismutase, glutathione reductase, glutathione peroxidase	
		(v) Reduced malondialdehyde level	
		(vi) Reduced the number of apoptotic cells	
		(vii) Suppressed the cleaved of caspase 3	
		(viii) Decreased the Bax/Bcl-2 ratio	

Boswellic acids	Rotenone-induced rats	(i) Increased motor functions	[240]
		(ii) Ameliorated percent of degenerating neuronal in the substantia nigra pars compacta	
		(iii) Increased percent of viable neurons in the substantia nigra pars compacta	
		(iv) Reduced inflammatory markers (TNF-*α*, IL-6, COX-2)	
		(v) Decreased NF-*κ*B level	
		(vi) Increased striatal dopamine level	
		(vii) Increased nigral tyrosine hydroxylase immunostaining	

*Capsicum annuum* L. extract	Rotenone-induced mouse	(i) Inhibited the increase of brain malondialdehyde and nitric oxide levels	[241]
		(ii) Restored brain GSH level and paraoxonase-1 (PON1) activity	
		(iii) Attenuated the increase in brain 5-lipoxygenase activity	
		(iv) Restored brain cholinesterase activity	
		(v) Decreased GFAP-positive immunoreactivity in the cerebral cortex	
		(vi) Prevented the neuronal degeneration in the substantial nigra, cerebral cortex, and hippocampus	

*Coeloglossum viride* var. bracteatum extract	MPTP-induced neurotoxicity in mouse and glutamate-induced excitotoxicity in primary cortical neuron cultures	(i) Inhibited glutamate-induced excitotoxicity in vitro	[242]
		(ii) Inhibited glutamate-induced in the decrease of phosphorylated Akt and Bcl-2	
		(iii) Prevented dopaminergic neuronal loss	

Curcuminoids (*Curcuma longa* (L.) rhizomes)	MPTP-induced mouse	(i) Prevented the depletion of dopamine and tyrosine hydroxylase immunoreactivity	[243]
		(ii) Reversed GFAP and iNOS protein expressions	
		(iii) Reduced proinflammatory cytokine and total nitrite generation in the striatum	
		(iv) Improved motor performance and gross behavioral activity, as determined by rotarod and open field tests	

*β*-Caryophyllene, a plant-derived cannabinoid compound known as phytocannabinoid	Rotenone-induced rats	(i) Rescued dopaminergic neurons	[244]
		(ii) Prevented dopaminergic neuronal loss in the substantia nigra and striatal dopamine fibers	
		(iii) Reduced Iba-1 and GFAP expressions	
		(iv) Decreased the number of activated astrocytes and microglia	
		(v) Attenuated proinflammatory cytokines (IL-1b, IL-6, and TNF-*α*) in the midbrain tissues and inflammatory mediators (COX-2 and iNOS expressions) in the cytoplasmic fraction of striatal tissue lysates	
		(vi) Restored antioxidant enzymes and glutathione depletion	
		(vii) Inhibited lipid peroxidation	

Fish oil supplementation (rich in omega-3 polyunsaturated fatty acids)	6-OHDA-induced rats	(i) Mitigated the loss of substantia nigra neurons and nerve terminals in the striatum	[245]
		(ii) Reduced the density of iNOS-immunoreactive cells and microglia (OX-42) and astrocyte (GFAP) reactivity	

Germinated brown rice	Rotenone-induced rats	(i) Enhanced the motor activity in rotenone-induced rats	[246]
		(ii) Decreased serum and brain TNF-*α*, dopaminergic neuronal loss, motor deficits, the percentage of apoptotic cells	
		(iii) Attenuated the dopaminergic neuronal cell loss	
		(iv) Attenuated histopathological changes in substantia nigra neurons with visible nuclei and	
		(v) Increased the number of surviving dopaminergic neurons	
		(vi) Decreased the number of apoptotic cells	
		(vii) Increased the number of viable cells	
		(viii) Decreased TNF-*α* level in the serum and in brain	

*Oxalis corniculata* extract	MPTP-induced mouse	(i) Improved memory retention and retrieval	[247]

Olive leaf extract (*Olea europaea* L.)	Rotenone-induced rats	(i) Suppressed oxidative stress by decreasing lipid peroxidation level and increasing midbrain antioxidant enzymes activities	[248]
		(ii) Inhibited the depletion of tyrosine hydroxylase-positive neurons	

Puerarin (an active component of *Pueraria montana* var. lobata (willd.) Sanjappa & Pradeep)	MPTP-induced mouse	(i) Attenuated MPTP-induced behavioral deficits, dopaminergic neuronal degeneration, and dopamine depletion	[249]
		(ii) Enhanced glutathione activity, glial cell line-derived neurotrophic factor (GDNF) expression, and PI3K/Akt pathway activation, which might ameliorate MPTP injection-induced progressive elevation of ROS formation in mouse	
		(iii) Ameliorated MPTP-reduced lysosome-associated membrane protein type 2A (Lamp 2A) expression	

Rosmarinic acid isolated from callus of *Perilla frutescens*	6-OHDA-induced rats	(i) Restored the striatal dopamine level	[250]
		(ii) Increased the number of tyrosine hydroxylase	
		(iii) Decreased the iron level in the substantia nigra	
		(iv) Upregulated the ratio of Bcl-2/Bax gene expression in the substantia nigra	

*Sophora tomentosa* extract	MPTP-induced mouse	(i) Alleviated MPTP-induced motor deficits	[251]
		(ii) Attenuated the decrease in the number of tyrosine hydroxylase-positive neurons in the substantia nigra	
		(iii) Restored the depletion of striatal dopamine level	
		(iv) Restored GSH level and antioxidant enzyme activities and decreased lipid peroxidation in the striatum	
		(v) Decreased the expression of *α*-synuclein and GSK-3*β* phosphorylation in the striatum	

*Tinospora cordifolia* ethanol extract	6-OHDA-induced rats	(i) Increased the dopamine levels and complex I activity	[252]
		(ii) Attenuated iron asymmetry ratio	
		(iii) Reduced oxidative stress	
		(iv) Restored 6-OHDA-induced behavioral changes in locomotor activity	
		(v) Reduced the degree of catalepsy	
		(vi) Increased the time of fall in rotarod test	

*Tribulus terrestris* extract	Rotenone-induced mouse	(i) Ameliorated motor dysfunction	[253]
		(ii) Increased the percentage of viable neurons	
		(iii) Increased the number of tyrosine hydroxylase	
		(iv) Attenuate inflammatory markers (iNOS and COX-2 mRNA expression)	
		(v) Reduced DNA damage markers (8-oxo-2′-deoxyquanosine and MTH1 expression)	
		(vi) Suppressed oxidative stress by increasing GSH and activities of superoxide dismutase and catalase and decreasing malondialdehyde level	
		(vii) Downregulated CD11b mRNA expression (microglia marker)	
		(viii) Improved striatal dopamine level	

Ethyl acetate fraction of *Urtica dioica* linn.	MPTP-induced rats	(i) Improved the motor function and oxidative defense alteration	[254]
		(ii) Decreased the increased concentration of lipid peroxidation and nitrite concentration	
		(iii) Restored the decreased GSH level and activity of catalase	
		(iv) Attenuated the proinflammatory cytokines (TNF-*α* and IL-*β*)	
		(v) Restored the level of dopamine and its metabolites	
		(vi) Protected the dopaminergic neurons	

*Zingiber zerumbet* (L.) Smith ethyl acetate extract	Paraquat-induced rats	(i) Decreased lipid peroxidation and protein oxidation	[255]
		(ii) Increased level of GSH and the activities of antioxidant enzymes	
		(iii) Prevented neuronal damage	

**Table 2:** Natural products and their bioactive compounds with neuroprotective activities in treating Alzheimer's disease.

Plant extracts/phytochemicals (plant source)/natural products/substances	Study model	Neuroprotective activities	References
Turmeric (powdered rhizome of *Curcuma longa* Linn (5% curcumin)	Case studies of 3 patients with progressive dementia	(i) Improvement in the behavioral symptoms and quality of life	[256]

Coconut oil enriched Mediterranean diet	44 patients with Alzheimer's disease	(i) improved the cognitive functions	[257]

Germinated brown rice (Malaysian mixed varieties; MR219 and MR220)	A*β* (1–42)-induced toxicity in SH-SY5Y cells	(i) Reduced intracellular ROS generation	[258]
		(ii) Attenuated A*β* (1–42)-induced cell death	

Huperzine A isolated from *Huperzia serrata*	Hypoxic-ischemic and glutamate-induced brain injury and cytotoxicity	(i) Reduce A*β* (1–42)-induced neuronal cell death	[259]
		(ii) Decrease oxidative damage	
		(iii) Protects neurons from cytotoxic and apoptosis	
		(iv) Inhibited the glutamate toxicity	

Huperzine A isolated from *Huperzia serrata*	50 patients with Alzheimer's disease	(i) Improvement in memory, cognitive, and behavior functions	[260]

Methanolic extract of *Lactuca capensis* thunb. leaves	A*β* (1–42)-induced neurotoxicity in rats	(i) Ameliorated cognitive impairment and memory deficits	[261]
		(ii) Decreased acetylcholinesterase activity	
		(iii) Restored the level of GSH and the activities of antioxidant enzymes (superoxide dismutase and glutathione peroxidase)	
		(iv) Decreased the lipid peroxidation and protein oxidation level	
		(v) Attenuated hippocampal apoptosis by lowering the enrichment factor of apoptosis level	
		(v) Increased BDNF mRNA copy number	
		(vi) Decreased IL-1*β* mRNA copy number	

Osmotin, a plant protein extracted from *Nicotiana tabacum*	A*β* (1–42)-treated mouse and A*β* (1–42)-induced neurotoxicity in HT22 cells	(i) Reversed synaptic deficits	[262]
		(ii) Attenuated A*β* accumulation and BACE-1 expression	
		(iii) Increased spontaneous alternation behavior	
		(iv) Ameliorated memory impairment in a *Y*-maze test	
		(v) Alleviated the hyperphosphorylation of the tau protein at serine 413 through the regulation of the aberrant phosphorylation of p-PI3K, p-Akt (serine 473), and p-GSK3*β* (serine 9)	
		(vi) Prevented A*β* (1–42)-induced apoptosis and neurodegeneration in the A*β* (1–42)-treated mouse	
		(vii) Attenuated A*β* (1–42)-induced neurotoxicity in vitro of neuronal HT22 cells and primary cultures of hippocampal neurons	

Safflower yellow (natural safflower aqueous extract)	A*β* (1–42)-induced rats	(i) Improved short- and long-term memory of rats	[263]
		(ii) Decreased inflammatory markers (iNOS, IL-1*β*, IL-6, and TNF-*α* levels)	
		(iii) Reduced neuronal cell loss in the hippocampus and cortex	
		(iv) Inhibited the activation of glial cells	
		(v) Downregulated M1 microglial markers (iNOS and CD86)	
		(vi) Upregulated M2 microglial markers (arginase-1, CD2066, and YM-1)	

*Tabernaemontana divaricata* root extract	A*β* (25–35)-induced mouse	(i) Prevented memory loss	[264]
		(ii) Decreased lipid peroxidation	
		(iii) Increased neuronal density in the hippocampus	

Yacon (*Smallanthus sonchifolius* (poepp. and endl.) H. Robinson) leaf extract	A*β* (25–35)-induced rats	(i) Decreased oxidative stress in the hippocampus	[265]
		(ii) Prevented memory deficits	
		(iii) Attenuated the hippocampal damage	

**Table 3:** Natural products and their bioactive compounds with neuroprotective activities in treating amyotrophic lateral sclerosis and multiple sclerosis as well as chronic inflammation.

Plant extracts/phytochemicals (plant source)/natural products/substances	Study model	Neuroprotective activities	References
Anthocyanin extracted from strawberries	G93A mutant human SOD1 (hSOD1^G93A^) mouse model of amyotrophic lateral sclerosis	(i) Delayed the onset of disease and extend survival of hSOD1^G93A^ mouse	[266]
		(ii) Preserved hind limb grip strength in the hSOD1^G93A^ mouse	
		(iii) Reduced astrogliosis (GFAP) in the spinal cord of hSOD1^G93A^ mouse	
		(iv) Preserved neuromuscular junctions in gastrocnemius muscle tissue	

*Alpinia oxyphylla* fruit extract	Experimental autoimmune encephalomyelitis mouse model of multiple sclerosis	(i) Reduced the symptoms in the experimental autoimmune encephalomyelitis mouse	[267]
		(ii) Reduced demyelination in the spinal cord	
		(iii) Reduced inflammation (IFN-*γ* and IL-17) in the spinal cord	
		(iv) Reduced gliosis in the spinal cord	
		(v) Alleviated T helper (Th)1/Th17 response	
		(vi) Reduced immune cell infiltration into spinal cord and brain	

Isogarcinol extracted from *Garcinia mangostana* L. mangosteen	Experimental autoimmune encephalomyelitis-induced mouse	(i) Alleviated inflammation and demyelination in the brain and spinal cord	[268]
		(ii) Ameliorated the clinical signs of experimental autoimmune encephalomyelitis-induced mouse	
		(iii) Reduced intracranial lesions	
		(iv) Reduced number of Th1 and Th17 cells differentiation by inhibiting Janus kinase (JAK)/signal transducers and activators of transcription (STAT) signaling pathway	
		(v) Reduced activation of CD4^+^ and CD11b^+^ cell populations	

*Ishige okamurae*	Experimental autoimmune encephalomyelitis-induced rats	(i) Reduced inflammatory markers (TNF-*α* and COX-2)	[269]
		(ii) Ameliorated the clinical signs of experimental autoimmune encephalomyelitis-induced rats	
		(iii) Suppressed T-cell proliferation	
		(iv) Ameliorated experimental autoimmune encephalomyelitis-induced paralysis	

*Nigella sativa*	Experimental autoimmune encephalomyelitis-induced rats	(i) Decreased transforming growth factor beta-1 (TGF-*β*1) expression	[270]
		(ii) Enhanced remyelination in the cerebellum	
		(iii) Suppressed inflammation	

*Radix rehmanniae* extract	Experimental autoimmune encephalomyelitis-induced mouse	(i) Reduced inflammation and demyelination in spinal cords	[271]
		(ii) Reduced CD3^+^ and CD11b^+^ cell populations in the spinal cord and brain	
		(iii) Ameliorated the clinical signs	
		(iv) Inhibited NF-*κ*B signaling	
		(v) Reduced expression of iNOS and NADPH oxidase	
		(vi) Reduced peroxynitrite level in spinal cords	

White grape (*Vitis vinifera*)	Experimental autoimmune encephalomyelitis-induced mouse	(i) Reduced TNF-*α*, iNOS, and PARP expression	[272]
		(ii) Reduced nitrotyrosine level	
		(iii) Inhibited apoptosis (caspase-3 and Bcl-2 expression)	
		(iv) Decreased the number of terminal deoxynucleotidyl transferase dUTP nick end labeling (TUNEL)-positive	
		(v) Modulated transcription factor Fork head box P3	

Walnut extract	Lipopolysaccharide-induced neurotoxicity in rat microglial cell line	(i) Downregulated iNOS and Iba-1 expressions	[273]
		(ii) Upregulated calmodulin expression	

**Table 4:** Natural products and their bioactive compounds with neuroprotective activities in treating other neurodegenerative diseases and neurological disorders.

Plant extracts/phytochemicals (plant source)/natural products/substances	Study model	Neuroprotective activities	References
Ethanolic extract of *Cocculus laurifolius* leaves	Strychnine-induced convulsions in albino rats	(i) Exhibited anticonvulsant activity by delaying the onset convulsion	[274]
		(ii) Maintained the structure of neurons	
		(ii) Decreased neuronal apoptosis	

*Coeloglossum viride* var. *bracteatum*	A combination of D-galactose and aluminum chloride-induced aging mouse	(i) Improved learning and memory in aging mouse	[275]
		(ii) Upregulated mRNA expression of BDNF and fibroblast growth factor 2 (FGF2) in the hippocampus of aging mouse	
		(iii) Inhibited mRNA expression of neuroinflammatory factors (TNF-*α*, IL-6, IL-1*β*, and NOS-2) in the hippocampus of aging mouse	
		(iv) Activated PI3K/Akt signaling pathway	
		(v) Inhibited the canonical caspase-3 apoptosis pathways	

Methanolic extract of C*innamomum camphora* leaves	Maximal electroshock-induced seizures in albino Wistar rats	(i) Exhibited the anticonvulsant activity in maximal electroshock-induced seizures by reducing epileptic seizures	[276]
		(ii) Increased gamma-aminobutyric acid (GABA) release	
		(iii) Decreased lipid peroxidation and acetylcholinesterase activity	
		(iv) Increased GSH level	

*Phragmanthera austroarabica extract*	Pentylenetetrazol-kindled mouse	(i) Reduced seizures and cortical malondialdehyde level	[277]
		(ii) Enhanced cortical GSH	
		(iii) Reduced the percentage of pyknotic neurons in the hippocampus	
		(iv) Increased the percentage of viable neurons	

Parawixin 10, a compound isolated from *Parawixia bistriata* spider venom	A rat excitotoxicity model of brain injury by kainic acid, N-methyl-D-aspartate, and pentylenetetrazol	(i) Decreased glial proliferation in all hippocampal subfields studied, as well as the preservation of cell layers	[278, 279]
		(ii) Prevented the onset of seizures induced with kainic acid, N-methyl-D-aspartate, and pentylenetetrazol	
		(iii) Increased the latency to the onset of kainic acid-, pentylenetetrazol-, and N-methyl-D-aspartate-induced seizures	

White rose (*Rosa hybrida*) petal extract	Kainic acid-induced mouse and in HB1.F3 human neural stem cells	(i) Exhibited radical scavenging activities	[280]
		(ii) Inhibited lipid peroxidation	
		(iii) Decreased scores of epileptiform seizures	
		(iv) Decreased hippocampal pyramidal neuronal loss	
		(v) Downregulated mRNA expressions of antioxidant enzymes	
		(vi) Downregulated mRNA and protein expressions of inflammatory mediators	

Rosemary extract	Kainic acid-induced rats	(i) Decreased neuronal loss in CA3 hippocampal region	[281]
		(ii) Decreased spatial memory and learning impairment	

Walnut extract	Lipopolysaccharide-induced neurotoxicity in rat microglial cell line	(i) Downregulated iNOS and Iba-1 expressions	[273]
		(ii) Upregulated calmodulin expression	

Taken together, these findings proposed that the natural products and their bioactive compounds may have a potential neuroprotective effect against neurodegenerative disease through various mechanisms, primarily through their antioxidant, anti-inflammatory, and antiapoptotic. This signifies the therapeutic evidence of the natural products and their bioactive compounds as neuroprotective agents.
